# Obesity alters the fitness of peritumoral adipose tissue, exacerbating tumor invasiveness in renal cancer through the induction of *ADAM12* and *CYP1B1*

**DOI:** 10.1002/1878-0261.13782

**Published:** 2025-01-13

**Authors:** Sepehr Torabinejad, Caterina Miro, Annarita Nappi, Francesco Del Giudice, Annunziata Gaetana Cicatiello, Serena Sagliocchi, Lucia Acampora, Federica Restolfer, Melania Murolo, Emery Di Cicco, Federico Capone, Ciro Imbimbo, Monica Dentice, Felice Crocetto

**Affiliations:** ^1^ Department of Clinical Medicine and Surgery University of Naples “Federico II” Italy; ^2^ Department of Neurosciences, Reproductive Sciences and Odontostomatology University of Naples “Federico II” Italy; ^3^ CEINGE – Biotecnologie Avanzate S.c.a.r.l. Naples Italy

**Keywords:** ADAM12, cancer‐associated adipocytes, CYP1B1, obesity, renal cell carcinoma

## Abstract

Obesity exacerbates the risk and aggressiveness of many types of cancer. Adipose tissue (AT) represents a prevalent component of the tumor microenvironment (TME) and contributes to cancer development and progression. Reciprocal communication between cancer and adipose cells leads to the generation of cancer‐associated adipocytes (CAAs), which in turn foster tumor invasiveness by producing paracrine metabolites, adipocytokines, and growth factors. Interfering with the crosstalk between CAAs and cancer cells is of key relevance in the prevention of tumor progression. The present study aimed to analyze the contribution of peritumoral AT in renal cell carcinoma (RCC) progression in lean versus overweight or obese patients. By isolating human adipose‐derived stromal/stem cells from the three groups of patients and performing conditioned medium studies with RCC cells along with *in vivo* xenograft experiments, we found that peritumoral adipocytes from the three groups show a distinct expression profile of genes. In particular, ADAM metallopeptidase domain 12 (*ADAM12*) and cytochrome P450 family 1 subfamily B member 1 (*CYP1B1*) were found to be upregulated in obesity and their silencing reduced RCC cell invasiveness. In conclusion, high *ADAM12* and *CYP1B1* expressions in the peritumoral adipocytes boost tumor invasiveness and may serve as an indicator of poor prognosis in RCC.

## Graphical abstract

Tumor microenvironment drives cancer formation and progression. We analyzed the role of human cancer‐associated adipocytes from patients with renal cell carcinoma (RCC) stratified as lean, overweight, or obese. RNA‐seq demonstrated that, among the most altered genes involved in the tumor–stroma crosstalk, are ADAM12 and CYP1B1, which were proven to be key promoters of RCC tumorigenesis. These findings add insight into fat‐driven cancerogenesis.

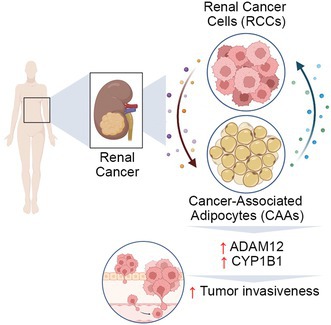

**Abbreviations**
*ADAM12*
: ADAM metallopeptidase domain 12
ASCs: adipose‐derived stromal/stem cells
AT: adipose tissue
BMI: body mass index
CAAs: cancer‐associated adipocytes
ccRCC: clear cell renal cell carcinoma
CM: conditioned medium
*CYP1B1*
: cytochrome P450 family 1 subfamily B member 1
DEGs: differentially expressed genes
GO: gene ontology
RCC: renal cell carcinoma
TME: tumor microenvironment

## 1 Introduction

Renal cell carcinoma (RCC) arises from renal tubular epithelial cells and encompasses 85% of all primary renal neoplasms. Smoking, obesity, chronic analgesic use, hypertension, and diabetes are the most common risk factors for this cancer. RCC is the fifth most common cancer and leads to a high rate of mortality [1]. RCC has several subtypes, among them clear cell RCC (ccRCC) is the most prevalent (75%). Ninety‐five percent of patients with ccRCC have lost the short arm of chromosome 3 containing the *VHL* tumor suppressor gene [2]. Additional alterations including mutation in the *PBRM1*, *SETD2*, and *BAP1* genes have also been identified in ccRCC [3]. Among ccRCC risk factors, obesity is of much importance, as it renders adipose tissue (AT) inflammation and may even trigger cancer initiation [4]. Indeed, since leptin secretion is proportional to the extent of AT and, in turn, leptin increases tumor growth and invasiveness in different cancer types [5, 6], including renal cancer [7], elevated incidence of obesity parallels higher risks of kidney cancer deaths.

AT is the primary site of excess energy storage and an endocrine organ that releases soluble factors to maintain homeostasis. However, as a part of the tumor microenvironment (TME), AT can lead to tumor progression. *In vitro* studies have shown that breast cancer cells receiving conditioned medium (CM) from adipose cultures or co‐cultured with adipocytes express high levels of inflammatory molecules [8, 9], have different protein profiles [10], and convert into invasive phenotype [8, 9, 11]. The supportive effect of AT on lung cancer has also been observed in both *in vitro* and *in vivo* experiments [12].

Crosstalk between cancerous cells and adjacent AT is bidirectional. Therefore, in addition to affecting cancerous cells, adipocytes undergo changes regulated by cancer cells [13, 14]. Wnt3a and TNFα molecules secreted by tumor cells reprogram adipocytes toward a de‐differentiated phenotype [15] so that they express stem cell markers and gain proliferative capacity [16, 17, 18]. Cancer cells also induce lipolysis and glycolysis in adipocytes that favor adipocyte de‐differentiation. Moreover, the metabolites produced through lipolysis (free fatty acid) and glycolysis (lactate) provide tumor energy requirements to grow and invade the adjacent tissues [19, 20, 21, 22]. In general, the alterations induced in tumor‐adjacent AT benefit tumor progression.

The results of the studies performed on kidney tumors confirm the AT alterations induced by tumor cells. For instance, influenced by kidney tumors, adjacent ATs underwent a browning process to increase tumor nutrients and facilitate their growth and invasion [1]. Furthermore, the ATs derived from human cancerous kidneys expressed higher levels of versican, leptin, leptin receptor, and perilipin 1 molecules compared to those extracted from normal kidneys. ATs around kidney tumors also decreased the cellular adhesion of RCC cell lines and increased their migration capability, whereas such effects were not observed in RCC cells influenced by normal kidneys' AT [23].

In the present study, we aimed to compare the mRNA expression profile of tumor‐adjacent ATs derived from RCC obese and nonobese patients. Among the differentially expressed genes, *ADAM12* (Disintegrin and Metalloproteinase 12) and *CYP1B1* (Cytochrome P450 Family 1 Subfamily B Member 1) were significantly different in obese compared to nonobese patients. Both these proteins can have a key role in fostering tumorigenesis. Indeed, ADAM12 is a Zn2^+^ dependent, pleiotropic protein, also involved in tumor formation and progression, besides being implicated in the pathogenesis of liver fibrosis, hypertension, and asthma [24]. CYP1B1 is a critical enzyme responsible for the regulation of endogenous metabolic pathways, including the metabolism of steroid hormones, fatty acids, and vitamins. Notably, since CYP1B1 catalyzes the activation of 7,12‐Di‐Methyl‐Benz[a] Anthracene (DMBA), Cyp1b1‐null mice have a drastic decreased frequency to develop malignant lymphomas after administration with DMBA compared to wild‐type mice [25]. Similarly, it was noted that CYP1B1 plays a key role in the metabolic activation of different environmental procarcinogens, suggesting that it may contribute to the maintenance of the homeostasis xenobiotic metabolism and as such, it can be of key relevance for cancer formation [26].

Therefore, using short‐hairpin RNAs (shRNAs), we silenced the expression of *ADAM12* and *CYP1B1* RNAs in obese/nonobese adipocytes. We observed that *ADAM12* and *CYP1B1* downregulation has a deep impact on the proliferative ability and the invasiveness of RCC cells, pointing to a critical role of these genes in regulating tumor–stroma interplay and fostering kidney cancer in obese patients.

## 2 Materials and methods

### 2.1 Patients and experimental groups

Thirty‐two RCC patients from February 2022 to March 2024 were enrolled in this study. They underwent nephrectomy surgery in the Group of Urology of the University Hospital Center ‘Federico II’, Naples, Italy. Written informed consents were collected before surgical procedures. The study methodologies conformed to the standards set by the Declaration of Helsinki. The study and protocols were approved by the ethical committee of the University of Naples ‘Federico II’ (prot. n. 118/20 of May 7, 2020). The patients were divided into three experimental groups based on their body mass index (BMI) including lean (18.5 ≤ BMI < 24.9 kg·m^−2^), overweight (25 ≤ BMI < 29.9 kg·m^−2^), and obese (BMI ≥ 30 kg·m^−2^) [27] (Table 1).

**Table 1:** Patients participating in the study. F, female; L, lean; M, male; Ob, obese; Ov, overweight; P, patient.

Patients	Age	BMI	Group	Gender
P1	73	36.33	Ob	M
P2	55	28.73	Ov	M
P3	64	27.16	Ov	M
P4	68	25.26	Ov	M
P5	74	42.28	Ob	M
P6	69	33.90	Ob	M
P7	69	25.08	Ov	M
P8	58	20.31	L	F
P9	76	22.70	L	M
P10	65	21.80	L	M
P11	69	31.10	Ob	M
P12	78	22.90	L	M
P13	38	40.00	Ob	M
P14	56	31.00	Ob	M
P15	57	27.00	Ov	M
P16	42	34.80	Ob	M
P17	59	21.30	L	F
P18	75	31.30	Ob	M
P19	42	20.00	L	F
P20	80	25.04	Ov	M
P21	49	32.70	Ob	M
P22	38	33.08	Ob	M
P23	53	23.00	L	F
P24	52	29.30	Ov	F
P25	46	26.30	Ov	M
P26	62	32.20	Ob	M
P27	66	18.50	L	M
P28	73	26.90	Ov	M
P29	74	31.60	Ob	M
P30	70	27.80	Ov	M
P31	60	20.50	L	M
P32	67	19.70	L	F

### 2.2 Sample preparation and adipose‐derived stromal/stem cell isolation

Following the surgery, biopsies of both the tumor and healthy kidneys were collected for molecular evaluation. In addition, 3–4 g of AT surrounding the RCC tumor were collected from each patient in order to isolate adipose‐derived stromal/stem cells (ASCs). First, the AT was cut into 3–4 mm pieces using a pair of sterile scissors. Then, the tissue was washed several times in Dulbecco's modified Eagle medium (DMEM, HiMedia Leading BioSciences Company, Mumbai, Maharashtra, India, Product Number: AL007‐500ML) containing 1% penicillin/streptomycin (Gibco, Thermo Fisher Scientific, Waltham, MA, USA, Product Number: 15070063) and digested with collagenase type IV at 37 °C (100 U·mL^−1^, Gibco, Thermo Fisher Scientific, Catalog Number: 17104‐019) according to the manufacturer's instructions. Following 4 h of digestion, the enzyme was inactivated by two volumes of DMEM+ 10% fetal bovine serum (FBS) (Gibco, Thermo Fisher Scientific). In the end, centrifugation of 1200 ***g*** for 10 min separated the stromal vascular fraction (SVF) containing ASCs as a pellet. Following filtering the SVF by a 70 μm cell strainer (Corning, Fisher Scientific, Hampton, NH, USA, Catalog Number: 431751), ASCs were seeded in 60‐mm diameter cell culture plates and cultured in normal growth medium (DMEM high glucose supplemented with 10% FBS, 2 mm L‐glutamine, 50 i.u. penicillin, and 50 μg·mL^−1^ streptomycin (all from Gibco, Thermo Fisher Scientific)) and at 37 °C in the presence of 5% CO_2_ (normal growth condition).

### 2.3 A498 and 786‐O cell lines

RCC cell lines (A498, RRID:CVCL_1056, and 786‐O, RRID:CVCL_1051), kindly provided by Prof. Angela Celetti (University of Naples, ‘Federico II’), were grown in DMEM and RPMI 1640 (HiMedia Leading BioSciences Company, Product Number: AL028), respectively, supplemented with 10% FBS, 1% penicillin/streptomycin, and 1% L‐glutamine, at 37 °C in the presence of 5% CO_2_. Cell lines were tested for mycoplasma contamination using the MycoAlert Plus assay (Lonza, Walkersville, MD, USA, Cat# LT07‐218) and were authenticated by short tandem repeat (STR) profiling.

### 2.4 Adipocyte differentiation

ASCs, cultured in growth medium, were induced for adipogenesis at passage three and at 80% confluence as previously described [28]. Briefly, adipocyte differentiation was initiated using the adipogenic medium containing growth medium supplemented with 1.0 μm Dexamethasone (Sigma‐Aldrich, St. Louis, MO, USA, Product Number: D4902), 0.5 mm 3‐Iso.Butyl‐1‐Methyl‐Xanthine (IBMX; Sigma‐Aldrich, Product Number: I7018), and 10.0 μg·mL^−1^ recombinant human insulin (Sigma‐Aldrich, Product Number: I2643). Following 48 h, the adipogenic medium was replaced with the maintenance medium (adipogenic medium lacking Dexamethasone and IBMX). Finally, the medium was changed every other day, until day 15 when the cells were differentiated into mature adipocytes.

### 2.5 Oil red O staining

The maturation of ASCs was evaluated by Oil Red O staining (Oil Red O, Sigma‐Aldrich, Catalog Number: O0625) as follows. After removing the culture medium from differentiated adipocytes and rinsing them with phosphate‐buffered saline (PBS), the cells were incubated at room temperature in formaldehyde solution 4%, pH 6.9 (Sigma‐Aldrich, Catalog Number: 1004968350) for 15 min. Then, formaldehyde was removed, and the adipocytes were rinsed with PBS. Next, 2 mL of 60% isopropanol was added, and the cells were incubated for 5 min at room temperature. Afterward, the isopropanol was removed, and 1 mL Oil Red O solution (200 μg·mL^−1^ in isopropanol) was added to each cell plate. Following 30 min of incubation with Oil Red O at room temperature, the cell plates were rinsed with tap water. The images were captured using a Leica DMi8 microscope and the Leica Application Suite LAS X Imaging Software (Leica Microsystems GmbH, Wetzlar, Germany).

### 2.6 mRNA sequencing for fully differentiated adipocytes

To compare the messenger RNA (mRNA) differential expression of the fully differentiated adipocytes between the three experimental groups, mRNA sequencing was performed for three samples of each group. First, total RNA (mRNAs) was extracted using TRIzol reagent (Life Technologies Ltd, Carlsbad, California, USA, Catalog Number: 15596018) and then purified and concentrated by RNeasy MinElute Cleanup kit (Qiagen, Hilden, Germany, Catalog Number: 74204) based on their manufacturers' protocols. Next, the total RNA was quantified using the Qubit 4.0 fluorometric Assay (Thermo Fisher Scientific). Libraries were prepared from 125 ng of total RNA using the NEGEDIA Digital mRNA seq research grade sequencing service (Next Generation Diagnostic srl, Pozzuoli, Italy). mRNA sequencing was performed by a NovaSeq 6000 sequencing system (Illumina Inc., San Diego, CA, USA) using a single‐end, 100‐cycle strategy. The raw data were analyzed after the cleaning step by quality filtering and trimming (bbduk, DOE Joint Genome Institute, Berkeley, CA, USA), alignment to the reference genome (hg38) using STAR 2.6.0a, counting by gene using HTseq‐counts 0.9.1, and normalized and visualized by Rosalind HyperScale architecture (OnRamp BioInformatics, Inc., San Diego, CA, USA).

### 2.7 Gene Ontology analysis and pathway enrichment analysis of DEGs

Gene Ontology (GO) and pathway enrichment analysis of newly identified differentially expressed genes (DEGs) were performed as reported in [19]. Stringent criteria were adopted for RNA‐seq data analysis: negatively regulated genes for the value of Log_2_ Fold‐Change (Log_2_ FC) ≤ −1.5, positively regulated genes for the value of Log_2_ Fold‐Change (Log_2_ FC) ≥ +1.5, with a false discovery rate (FDR) ≤ 0.05. To investigate the gene sets in the online biological network repository NDEx, the Molecular Signatures Database (MSigDB) was explored using the gene set enrichment analysis (gsea) software, a joint project of UC San Diego and Broad Institute (http://www.gsea‐msigdb.org/gsea/index.jsp). Furthermore, as confirmation, GO enrichment analysis was performed using also other different tools, as enrichr (https://maayanlab.cloud/Enrichr/) and metascape (https://metascape.org).

### 2.8 Short hairpin RNA‐mediated knockdown of *ADAM12* and *CYP1B1*

Cloning strategies for the generation of *ADAM12* and *CYP1B1* shRNA are reported elsewhere [17]. In brief, 2 × 10^6^ suspended adipocytes on day 7 of adipogenesis were plated in 60‐mm diameter plates coated with Matrigel (BD Biosciences, Palo Alto, CA, USA; Catalog Number: 354234) and then transfected with three different shRNA targeting endogenous *ADAM12* (shADAM12 #1, shADAM12 #2, and shADAM12 #3) or *CYP1B1* (shCYP1B1 #1, shCYP1B1 #2, and shCYP1B1 #3) or a scramble shRNA (shCTR) as a negative control using Lipofectamine‐3000 (Invitrogen™, Carlsbad, California, USA, Catalog Number: L3000015). shRNA‐targeted *ADAM12* and *CYP1B1* sequences were selected from the BLOCK‐iT™ RNAi Designer (Invitrogen™, Thermo Fisher Scientific, Carlsbad, California, USA). Oligos were designed and ordered from Eurofins Genomics. shRNA oligos were cloned into the EcoRI (New England Biolabs, Ipswich, Massachusetts, USA, Catalog Number: R0101) and XhoI (New England Biolabs, Catalog Number: R0146) sites of a pcRNAi plasmid. The cloning steps are schematically shown in Figs S1 and S2. 48 h after transfection, while the medium was collected for the next conditioned medium experiments, total protein lysate was collected and analyzed by western blot, and total RNA was extracted and analyzed by real‐time PCR.

### 2.9 Multiplex ELISA assay

Conditioned media (CMs) from *ADAM12*/*CYP1B1* expressing and silencing adipocytes of lean, overweight, and obese RCC patients were analyzed for the concentration of a panel of different cytokines and chemokines (IL‐1β, IL‐2, IL‐4, IL‐7. IL‐9, IL‐10, IL‐12 (P70), IL‐13, IL‐15, IL‐1ra, and PDGF‐bb) using custom Human Magnetic Luminex Assay (CUSTOM‐LXSA‐H‐21, R&D System, Austin, TX, USA), according to the manufacturer's protocol. The magnetic bead‐based multiplex assay was performed on a Luminex® 200™ System (Bio‐Techne, R&D Systems, Catalog Number: LX200‐XPON‐RUO). xPONENT software was utilized for the analysis of the results.

### 2.10 3D‐spheroid formation assay

Multicellular 3D‐spheroids were generated from 786‐O cells in Ultra‐Low Attachment surface plates. In brief, 786‐O cells were resuspended in normal growth medium and plated in an Ultra‐Low Attachment Polystyrene 96‐well (Corning™‐Costar®, Fisher Scientific, Catalog Number: 7007) at a final concentration of 5 cells per well and along with 25% CM. The Leica DMi8 microscope digital camera system and the Leica Application Suite LAS X Imaging Software (Leica Microsystems GmbH) were used to measure the diameter of the spheroids following 2, 4, 7, 9, and 11 days after seeding. DAPI Nucleic Acid staining (4′,6‐Diamidino‐2‐Phenylindole, Dihydrochloride, Invitrogen™, Carlsbad, California, USA, Catalog Number: D1306, Staining Condition: 1 : 1000 DAPI stock solution 2.5 mg·mL^−1^, 10 min directly in cell culture medium) was performed for the not‐fixed spheroids on the last day of the experiment.

### 2.11 Wound healing scratch assay

786‐O cells were seeded in 60‐mm diameter cell culture plates until reaching 100% confluence. The cells were then treated with a starvation medium (RPMI 1640 with 0.5% FBS, 1% Pen/Strep, and 1% L‐Glutamine, all from Gibco, Thermo Fisher Scientific) overnight. At Time Zero (T0), a straight‐line scratch was made on the monolayer of the confluent 786‐O cells using a sterile 200 μL pipette tip across the center of the plates. The detached cells were removed by washing with PBS, and then, the cells were treated with 50% CM from *ADAM12* or *CYP1B1* silenced adipocytes and 50% starvation medium. The images were taken at T0, 3 h, and 6 h after the treatment with CM using ×10 magnification with an inverted microscope (Leica DMi8, Leica Microsystems GmbH). The cell‐free area of each case was calculated by imagej (NIH Image, Bethesda, MD, USA) software considering the subtraction of the cell‐free surface of T0 from each endpoint [29].

### 2.12 Subcutaneous tumor xenograft model

Male NOD/SCID mice (6–8 weeks old) were purchased from Jackson Laboratory (Bar Harbor, ME, USA). Mice were housed in a controlled environment (20–22 °C, 12‐h light/dark cycle) with *ad libitum* access to food and water. The human renal cancer cell line (A498) was grown in DMEM (Microgem); supplemented with 10% FBS, 1% Pen/Strep, and 1% L‐Glutamine, at 37 °C in 5% CO_2_. Cells were harvested at 70–80% confluence using trypsin–EDTA. Nude mice were anesthetized with isoflurane, and 3 × 10^6^ RCC cells in 100 μL PBS were co‐injected with 3 × 10^6^ CAAs transfected with shCTR, shADAM12, or shCYP1B1. Postoperative analgesics (buprenorphine) were administered as needed. Tumor growth was measured twice weekly using calipers, and volumes were calculated using the formula (length × width^2^)/2. The study was terminated when tumors reached a volume of 2 cm^3^. Data were analyzed using (graphpad prism, Boston, MA, USA). Tumor volumes were compared using ANOVA, with a significance level set at *P* < 0.05. All animal procedures were approved by the Institutional Animal Care and Use Committee (IACUC, protocol n. 354/2019‐PR) from ‘Ministero della Salute’ (Department of Human Health, Animal Health and Ecosystem and International Relations).

### 2.13 Real‐time PCR

The total amount of mRNAs was extracted using TRIzol reagent (Life Technologies Ltd, Catalog Number: 15596018). Complementary DNAs (cDNAs) were synthesized using SuperScript™ VILO™ MasterMix (Life Technologies Ltd, cod. 11755‐050) starting from 1.0 μg of mRNA, according to the manufacturer's instructions. On CFX Connect Real‐Time PCR Detection System (Bio‐Rad, Hercules, CA, USA, cod. 1855201), real‐time PCR was directed using the fluorescent double‐stranded DNA‐binding dye SYBR Green (Bio‐Rad, cod. 1708882). All samples were analyzed in technical triplicate and standardized to an endogenous control, cyclophilin‐A (*CYPA*). The relative quantification (RQ) and the expression of each mRNA were determined utilizing the comparative Ct methodology and expressed as *N*‐fold differences in target gene expression $\left[N^{*}\text{target}=2^{\left(\Delta C_{t}\;\text{sample}-\Delta C_{t}\;\text{calibrator}\right)}\right]$. Specific primers for each gene were designed to work under the same cycling conditions [95 °C for 10 min > 40 cycles at 95 °C for 15 s > 60 °C for 1 min], thus generating amplicons of comparable sizes (100–300 bp). Primer combinations were placed whenever possible to span an exon–exon junction, and the total RNAs were digested with DNase to avoid genomic DNA contamination. Primer sequences are indicated in Table 2 [30].

**Table 2:** Oligonucleotide primers used in the study.

Gene	Forward sequence	Length	Reverse sequence	Length	Product length
CYPA	AGTCCATCTATGGGGAGAAATTTG	24	GCCTCCACAATATTCATGCCTTC	23	196, 195, 194
Leptin	GGCTTTGGCCCTATCTTTTC	20	GGATAAGGTCAGGATGGGGT	20	188
Adiponectin	CAGGCCGTGATGGCAGAGAT	20	GACCTTCAGCCCCGGGTACT	20	114
IL6	ATCCTCGACGGCATCTCAGC	20	CAAACTCCAAAAGACCAGTGAT	22	190
CXCR4	CTGGCCTTCATCAGTCTGGA	20	TCATCTGCCTCACTGACGTT	20	167
SDF1	CTTTCACTCTCCGTCAGCCG	20	TTGAGATGCTTGACGTTGGC	20	229
BAFFR	CACTGGTCCTGGCGCTGGTC	20	GTGGCATCAGAGATTCCCGG	20	160
ADAM12	TCGACTACAACGGGAAAGCA	20	CGTGTAATTTCGAGCGAGGG	20	130
CYP1B1	TCCTCCTCTTCACCAGGTATCC	22	GACAGGCACAAAGCTGGAGA	20	164
VHL	CTCCCAGGTCATCTTCTGCA	20	CTTGACTAGGCTCCGGACAA	20	301
PBRM1	CTTCTTTCACCGGCACTCAG	20	TGTGCGATGTAAGCCTGAGA	20	180
SETD2	AACAGAGTCAGCATCAGAGC	20	AACCATTAGCCGGGATAAGC	20	153
BAP1	CGACCTTCAGAGCAAATGTC	20	CTGGTGGGCAAAGAACATGT	20	157

### 2.14 Protein extraction and western blot analysis

Total protein was extracted using a lysis buffer (containing 0.5% Triton X‐100, Product Number: T8787; 25.0 mm Tris/HCL pH 7.4; 300 mm NaCl; and 1.0 mm CaCl_2_) supplemented with protease inhibitors including Protease Inhibitor Cocktail 1X, Product Number: P8340; 25.0 mm β‐Glycerophosphate disodium, Product Number: G5422; 1.0 mm Na_3_VO_4_, Product Number: S6508; 50.0 mm NaF, Product Number: 201154; and 1.25 mm PMSF; Product Number: P7626 (all from Sigma‐Aldrich). The total extracted protein was quantified by Protein Assay Dye Reagent (Bio‐Rad) using BSA as a standard. In the next step, 30 μg protein of each sample was run on a 10% SDS/PAGE gel and then transferred onto an Immobilon‐P transfer membrane (Millipore, Burlington, MA, USA, cod. GE 10600023). The membrane was blocked with bovine serum albumin (BSA) 5% (Sigma‐Aldrich) in PBS‐0.2% Tween at room temperature for 1 h. After the blocking step, the membrane was incubated in the primary antibody overnight at 4 °C. On the next day, following 1 h treatment of the membrane with the secondary antibody at room temperature, the specific protein bands were detected by chemiluminescent HRP substrate (Millipore, Catalog Number: WBKLS0500) and a Chemidoc XRS+ system (Bio‐Rad). The specific protein bands were quantified by imagej software (NIH Image). The primary antibodies used in this study were anti‐ADAM12 (1G3) (Santa Cruz Biotechnology, Dallas, TX, USA; cod. sc‐293 225, 1 : 1000) and anti‐CYP1B1 (G‐4) (Santa Cruz Biotechnology, cod. sc‐374 228, 1 : 1000). The secondary antibodies were anti‐mouse IgG‐HRP (Bio‐Rad, cod. 1706516, 1 : 3000) and anti‐rabbit IgG‐HRP (Bio‐Rad, cod. 1706515, 1 : 3000). In addition, anti‐beta actin (Cell Signaling Technology, Danvers, MA, USA, co. 8457S, 1 : 1000) or anti‐GAPDH (Elabscience, Houston, TX, USA, cod. E‐AB‐20059) were utilized as loading control antibodies [31].

### 2.15 Statistical analysis

In this study, all experiments were performed in triplicates, and data were described as mean ± standard deviation. In the case of two‐group comparisons, *T*‐student and for more than two‐group comparisons, one‐way or two‐way ANOVA tests were used to show the statistically significant differences between the groups (graphpad prism 9). *P*‐value (*P*) less than 0.05 was considered significant.

## 3 Results

### 3.1 Peritumoral adipocytes from RCC patients show alteration in leptin/adiponectin ratio and increase the expression of inflammatory genes according to the body weight

**Fig. 1:**
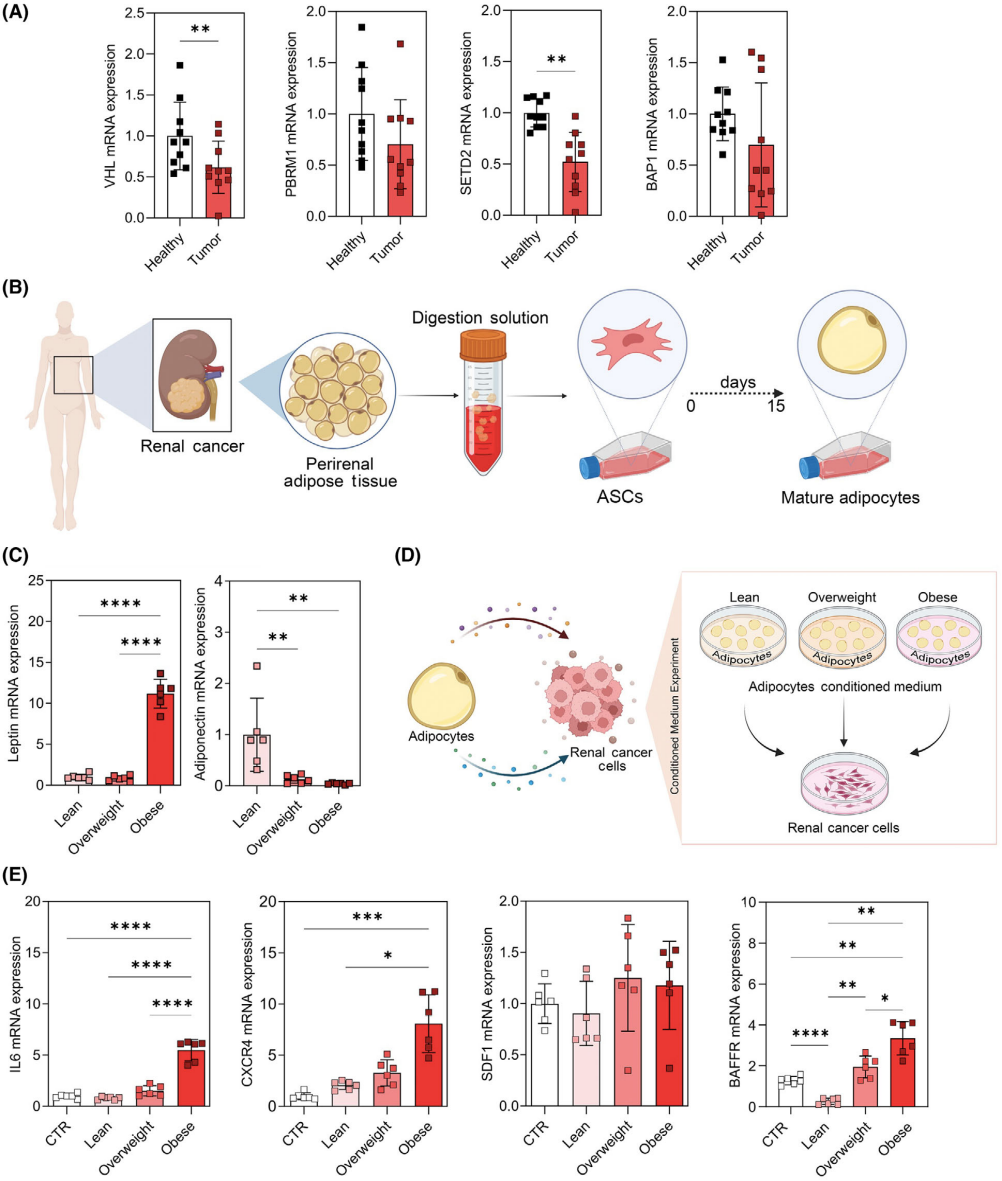
Adipose peritumoral tissue affects the expression of inflammatory genes by renal cell carcinoma (RCC) cells. (A) Relative mRNA expression of the different tumor suppressor genes in the healthy and tumor kidney tissues of RCC patients (*n* = 10), including *VHL*, *PBRM1*, *SETD2*, and *BAP1*, was measured by real‐time PCR. Cyclophilin‐A was used as an internal control. Data are shown as mean ± SD from at least 3 separate experiments. *P*‐values were determined by Student's *t*‐test. (B) Schematic figure illustrating the protocol for differentiation of adipocytes from peritumoral adipose tissue (AT) of lean, overweight, and obese RCC patients. (C) Relative mRNA expression of the leptin and adiponectin of the differentiated adipocytes among the lean, overweight, and obese groups (*n* = 6). Data are shown as mean ± SD from at least 3 separate experiments. *P*‐values were determined by one‐way ANOVA. (D) Schematic figure of the conditioned medium (CM) experiment, showing RCC cells treated with conditioned media (CMs) of lean, overweight, and obese differentiated adipocytes. (E) Relative mRNA expression of the *IL6*, *CXCR4*, *SDF1*, and *BAFFR* of the A498 cells treated with CMs collected from differentiated adipocytes derived from the lean, overweight, and obese groups (*n* = 6). Data are shown as mean ± SD from at least 3 separate experiments. *P*‐values were determined by one‐way ANOVA. *P < 0.05, **P < 0.01, ***P < 0.001, and ****P < 0.0001.

In order to characterize the differences in peritumoral adipose‐derived stromal/stem cells (ASCs), we collected a number of 32 RCC patients, stratified into three groups: lean (18.5 ≤ BMI < 24.9 kg·m^−2^, *n* = 10), overweight (25 ≤ BMI < 29.9 kg·m^−2^, *n* = 10), and obese (BMI ≥ 30 kg·m^−2^, *n* = 10), according to the BMI classification criteria established by World Health Organization (WHO) [27] (Table 1). We also collected the renal cancer tissue that was assessed for the expression of classical onco‐suppressor genes, downregulated in RCC, namely von Hippel–Lindau (*VHL*) Polybromo‐1 (*PBRM1*), *SETD2*, and *BAP1* [32, 33]. As expected, the expression of the onco‐suppressor genes was reduced in the tumors (Fig. 1A and Table 3), with no difference between the three classes of subjects (Fig. S3).

**Table 3:** The percentage of the patients with a downregulation in the mRNA expression of tumor suppressors in the RCC tumor vs healthy kidney tissue.

Gene	% downregulated patients	% downregulated patients (fold‐change ≥ −1.5)
VHL	90	70
PBRM1	80	50
SETD2	90	90
BAP1	70	70

We next isolated ASCs from the peritumoral human adipose tissue, that were cultured in an adipogenic condition for 15 days to become mature adipocytes (Fig. 1B and Fig. S4). We measured the expression of the two major adipocytokines (leptin and adiponectin) by real‐time PCR. The results showed that the expression of leptin was higher in obese adipocytes than in other groups while the expression of adiponectin was higher in lean adipocytes compared to the other experimental groups (Fig. 1C and Fig. S5). These results are consistent with the criteria of the mature lean and obese adipocytes and confirm both adipocyte maturation and the consistency of the adipocytes with the patients' BMI.

To test the influence of mature adipocytes from the lean, overweight, and obese patients on RCC cancer cells, we used the adipocytes conditioned medium (CM) to treat RCC cells (A498) for 48 h (Fig. 1D). Given the ability of AT to foster inflammation in the tumor–stroma, we first measured the expression of known inflammatory genes. As can be seen in Fig. 1E, obese CM could increase the expression level of the *IL6*, *CXCR4*, and *BAFFR* in A498 cells while it did not have any effect on the expression of the *SDF1*.

### 3.2 Obesity modifies the expression profile of mature adipocytes in the RCC tumor microenvironment

**Fig. 2:**
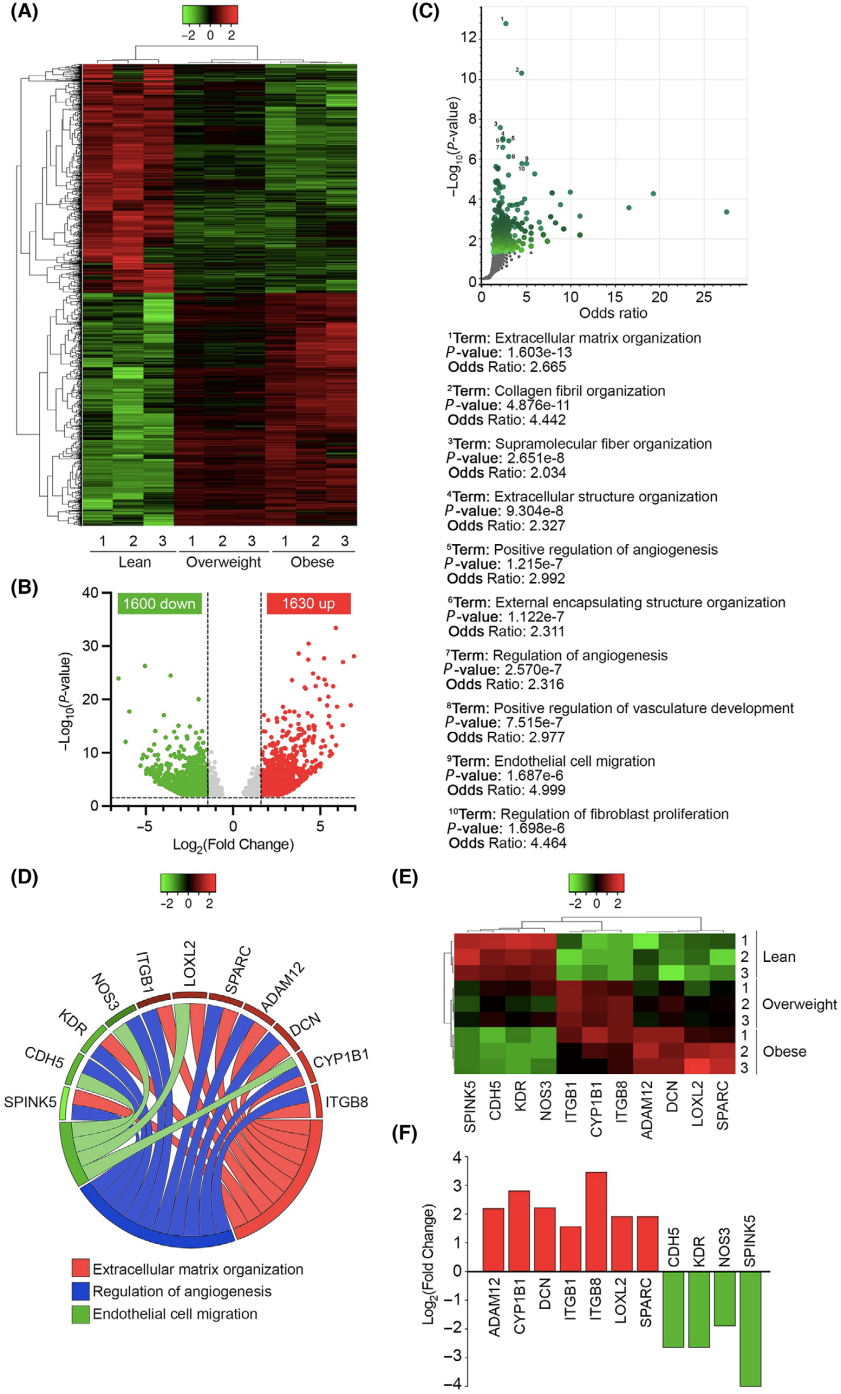
An increase in body mass index (BMI) induces a differential profile of gene expression in differentiated adipocytes from peritumoral adipose tissue (AT). (A) The heatmap (*n* = 3 patients for lean/overweight/obese groups) and (B) Volcano plot of the gene expression of the differentially expressed genes (DEGs) in the obese group (*n* = 3) in comparison with the overweight (*n* = 3) and lean (*n* = 3) groups; showing 1600 and 1630 upregulated and downregulated genes, respectively. In gray, are represented the genes that do not pass the parameters of the filters selected (|Fold‐Change| (FC): −1.5 ≥ FC ≥ + 1.5, *P*‐value < 0.05); in red, are represented the genes upregulated (FC ≥ + 1.5, *P*‐value < 0.05); and in green, are represented the genes downregulated (FC ≤ −1.5, *P*‐value < 0.05) in the obese group in comparison with the overweight and lean groups. The vertical dashed lines represent the Log_2_ Fold‐Change (Log_2_ FC) cut‐off values setted for the Gene Ontology and pathway enrichment analyses of DEGs. (C) Volcano plot of the molecular pathways which are different among the experimental groups; showing 10 top pathways based on the DEGs. (D) Selected molecular pathways and related DEGs for a close investigation along with the difference in the gene expression among the groups. (E) The heatmap and (F) bar chart of the DEGs related to the selected molecular pathways, indicating the expression difference among the groups.

In order to find the differentially expressed genes (DEGs) in the fully differentiated adipocytes of lean, overweight, and obese RCC patients, mRNA sequencing was performed. After analysis of the sequencing raw data, 3230 genes were detected as DEGs of which 1630 were overexpressed for the value of Log_2_ Fold‐Change (FC) ≥ +1.5 and 1600 were downregulated for the value of Log_2_ Fold‐Change (FC) ≤ −1.5 (both with *P* < 0.05) in the obese adipocytes in comparison with the other two groups (Fig. 2A,B). Based on Gene Ontology (GO) enrichment analysis in biological processes (BP), the top 10 molecular pathways related to the DEGs were detected (Fig. 2C). Among these pathways, three of them were selected for more focus: (a) extracellular matrix organization, (b) regulation of angiogenesis, and (c) endothelial cell migration (Fig. 2D). In the mentioned three pathways, 11 genes had a gradient expression (either upward or downward) from the lean to the overweight and from the overweight to the obese adipocytes (Fig. 2E,F).

### 3.3 *ADAM12* and *CYP1B1* genes are overexpressed in the peritumoral adipose tissue from obese patients compared to overweight and lean patients

**Fig. 3:**
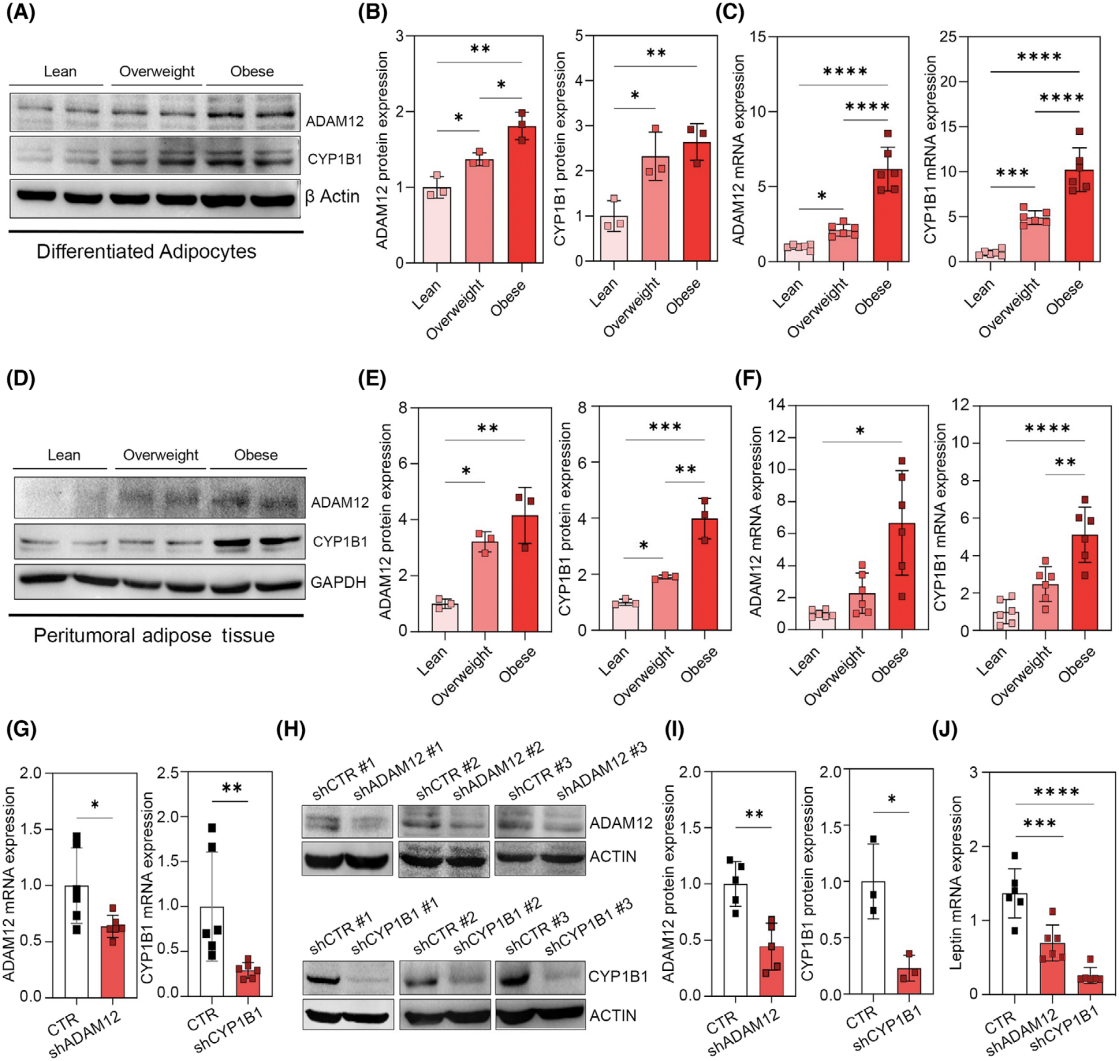
*ADAM12* and *CYP1B1* expression in peritumoral adipose tissue (AT) and differentiated adipocytes. (A, B) Protein (*n* = 3) and (C) mRNA expression (*n* = 6) of *ADAM12* and *CYP1B1* of 15‐day differentiated adipocytes among the lean, overweight, and obese groups. Data are shown as mean ± SD from at least 3 separate experiments. *P*‐values were determined by one‐way ANOVA. (D, E) protein (*n* = 3) and (F) mRNA (*n* = 6) expression of *ADAM12* and *CYP1B1* of peritumoral adipose tissue (AT) among the lean, overweight, and obese groups. (G) mRNA (*n* = 6) and (H, I) protein expression of *ADAM12* (*n* = 5) and *CYP1B1* (*n* = 3), after short‐hairpin RNA (shRNA)‐mediated silencing compared to the controls (adipocytes with scrambled RNA interference). Data are shown as mean ± SD from at least 3 separate experiments. *P*‐values were determined by Student's *t*‐test. (J) mRNA (*n* = 6) of *Leptin* after short‐hairpin RNA (shRNA)‐mediated silencing compared to the controls (adipocytes with scrambled RNA interference). Data are shown as mean ± SD from at least 3 separate experiments. *P*‐values were determined by one‐way ANOVA. *P < 0.05, **P < 0.01, ***P < 0.001 and ****P < 0.0001.

Among the DEGs in the three above‐mentioned pathways, *ADAM12* and *CYP1B1* are overexpressed in the obese group compared to the other groups and were selected for further studies due to their relevance in cancer [34, 35]. mRNA and protein quantification by real‐time PCR and western blot confirmed the mRNA sequencing results for these two genes not only in the differentiated adipocytes (Fig. 3A–C) but also in the relative adipose tissue (Fig. 3D–F). They show that differentiated adipocytes and the peritumoral adipose tissue from RCC patients with obesity have higher expression of *ADAM12* and *CYP1B1* genes and proteins compared to overweight and lean patients and suggest that the differential expression of these genes can be related to the ability of AT to worsen tumor invasiveness. To verify this hypothesis, we used three different specific short‐hairpin RNAs (shRNAs) for human *ADAM12* and *CYP1B1* to silence the protein expression of these two genes (Figs S1 and S2). 48 h after the adipocyte transfection with the shRNAs, *ADAM12*, and *CYP1B1* expressions were evaluated at the mRNA and protein levels by real‐time PCR and western blotting, respectively. shRNA treatments successfully decreased the expression level of *ADAM12* to 0.63% and 44% at mRNA and protein levels, respectively (Fig. 3G–I). Concerning *CYP1B1*, the decrease was even more as the expression level of this gene decreased to 30% at mRNA and 22% at protein level (Fig. 3G–I). Notably, the levels of leptin mRNA were drastically reduced in CAAs silenced for both, ADAM12‐ and CYP1B1 compared to control CAAs (Fig. 3J). This is consistent with the notion that *ADAM12* and *CYP1B1* are key genes in RCC tumorigenesis.

### 3.4 *ADAM12* and *CYP1B1* silencing alters the level of secretory molecules of adipocytes derived from RCC patients

**Fig. 4:**
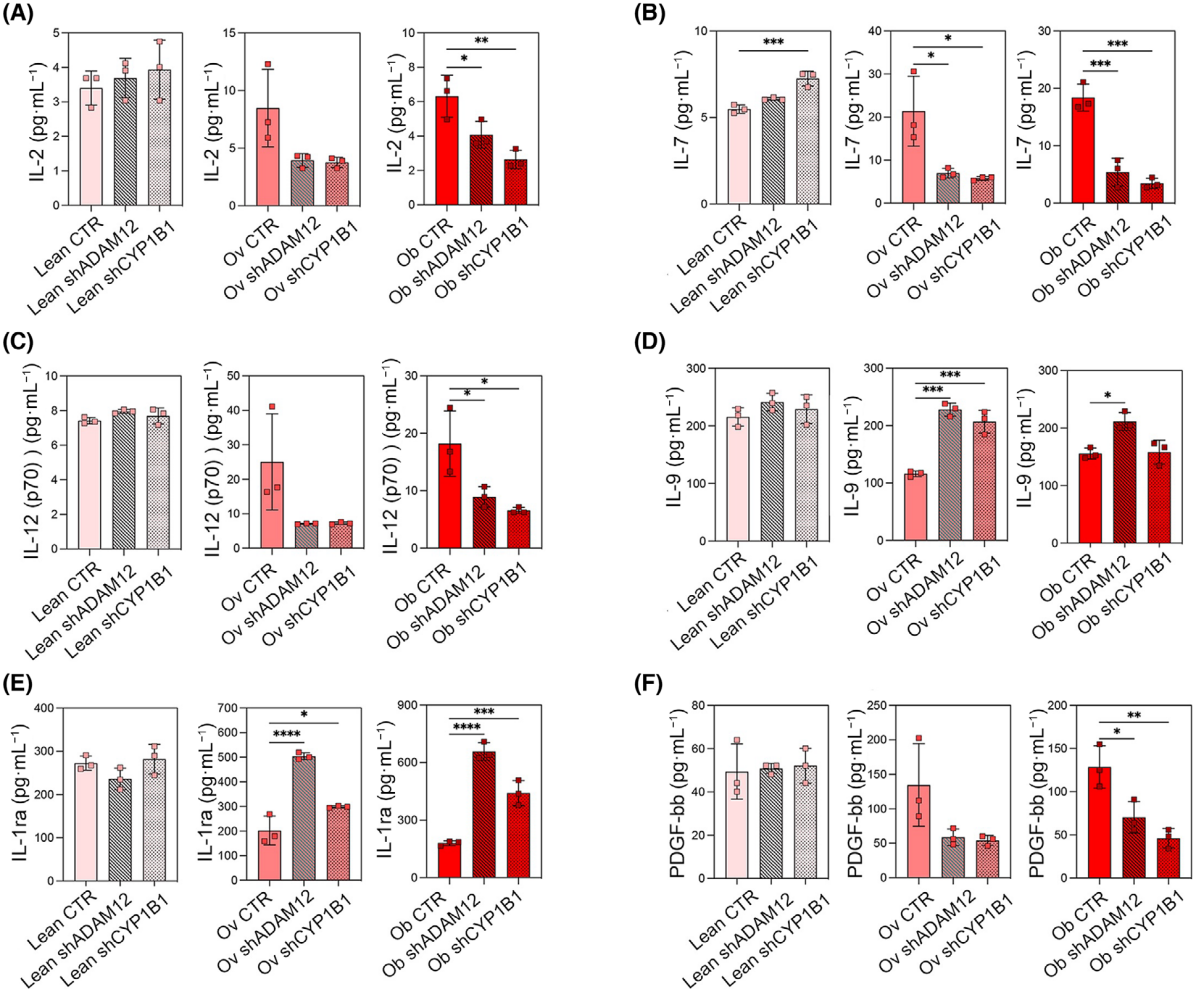
*ADAM12*/*CYP1B1* expression influences the secretory profile of differentiated adipocytes derived from renal cell carcinoma (RCC) patients. (A) IL‐2, (B) IL‐7, (C) IL‐12 (p70), (D) IL‐9, (E) IL‐1ra, and (F) PDGF‐bb concentrations (pg·mL^−1^) in conditioned media (CMs) of *ADAM12* and *CYP1B1* expressing and knocked down differentiated adipocytes derived from lean, overweight, and obese RCC patients (*n* = 3). CTR, CM from control adipocytes (adipocytes expressing *ADAM12* and *CYP1B1*); Ob, obese; Ov, overweight; shADAM12, CM from *ADAM12* silenced adipocytes; shCYP1B1, CM from *CYP1B1* silenced adipocytes. Data are shown as mean ± SD from at least 3 separate experiments. *P*‐values were determined by one‐way ANOVA. *P < 0.05, **P < 0.01 ***P < 0.001 and ****P < 0.0001.

To compare the differences between the concentration of soluble molecules in the conditioned media (CMs) of *ADAM12*/*CYP1B1* expressing (control) and silenced adipocytes of lean, overweight, and obese RCC patients, we performed multiplex ELISA assay. The pro‐inflammatory cytokines, IL‐2, IL‐7, and IL‐12 were downregulated by *ADAM12*/*CYP1B1* silencing in the overweight and obese‐derived adipocytes (Fig. 4A–C). Conversely, the anti‐tumor cytokine IL‐9 [36] and the anti‐inflammatory cytokine IL‐1ra were upregulated by *ADAM12*/*CYP1B1* silencing in the overweight and obese‐derived adipocytes (Fig. 4D,E). Also, the expression of PDGF‐bb, which is a promoter of cancer cell proliferation [37], was significantly reduced by suppression of *ADAM12* and *CYP1B1* in obese‐derived adipocytes (Fig. 4F). Other cytokines were not affected by the silencing as shown in Fig. S6. Interestingly, most of the effects of knocking down *ADAM12* and *CYP1B1* were observed in overweight and obese‐derived adipocytes, confirming the relevance of these two genes not only in tumorigenesis but also in the crosstalk between adipose tissue and cancer. This analysis reveals that the overexpression of *ADAM12* and *CYP1B1* is not only important for the tumorigenesis of RCC cells but also for the secretory profile of peritumoral adipocytes, thus opening new avenues for a deeper investigation of the ADAM12 and CYP1B1‐dependent CAAs secretome.

### 3.5 Conditioned media from *ADAM12* / *CYP1B1* silenced adipocytes reduces the proliferation of RCC cells

**Fig. 5:**
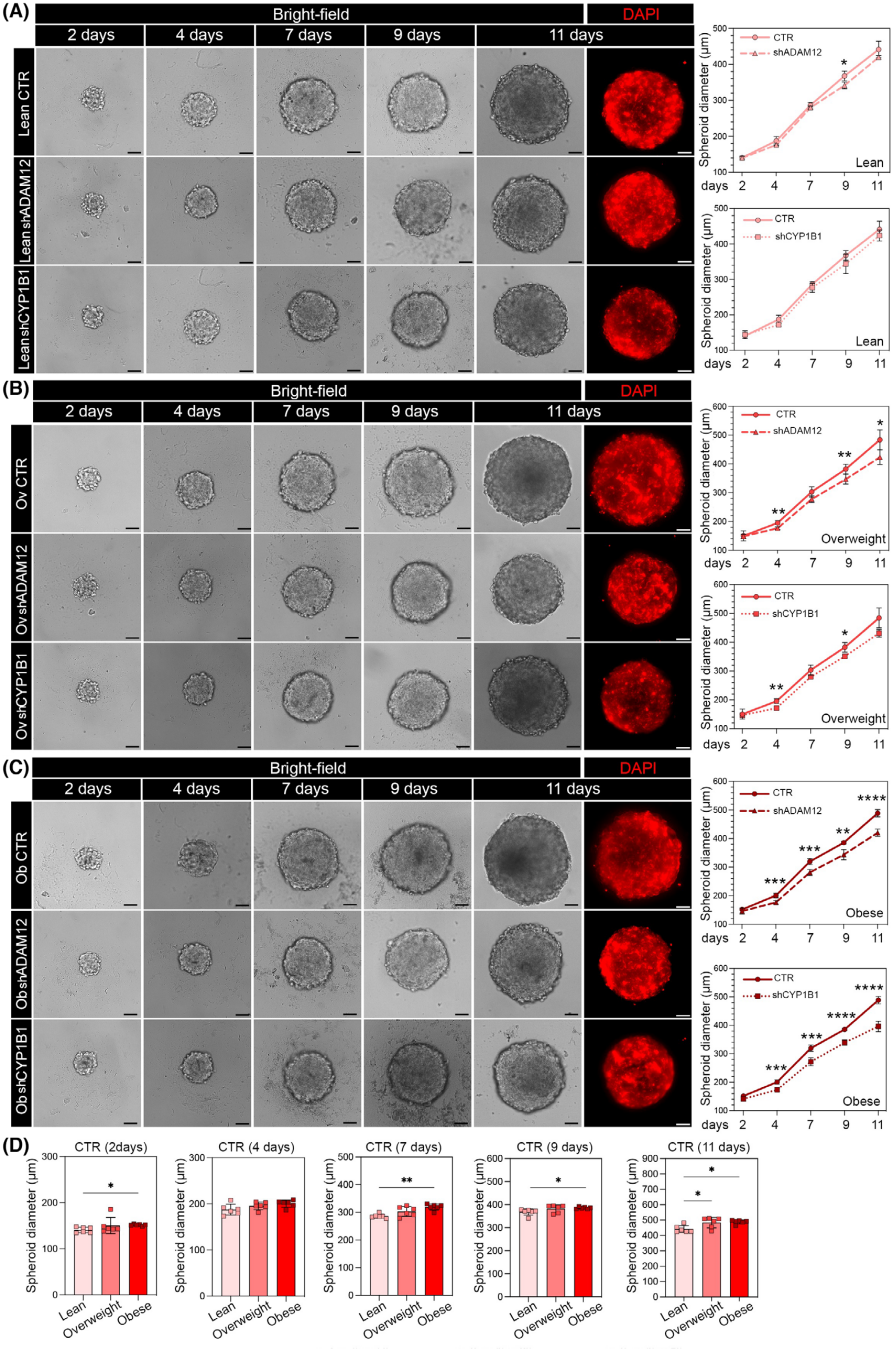
The knockdown of *ADAM12* and *CYP1B1* in adipocytes reduces the proliferation ability of renal cell carcinoma (RCC) cells. 3D‐Spheroids of 786‐O cells and their diameter measurements on 2, 4, 7, 9, and 11 days after treatment with conditioned media (CMs) derived from *ADAM12*/*CYP1B1* silenced and non‐silenced adipocytes of (A) lean, (B) overweight, and (C) obese RCC patients (*n* = 6). (D) Histograms show the spheroid diameter comparison in RCC cells treated with the CMs collected from *ADAM12*/*CYP1B1* expressing adipocytes of lean, overweight, and obese patients at the same time points as previously described (*n* = 6). CTR, 786‐O cells treated with CM from control adipocytes (adipocytes expressing *ADAM12* and *CYP1B1*); Ob, obese; Ov, overweight; shADAM12, 786‐O cells treated with CM from *ADAM12* silenced adipocytes; shCYP1B1, 786‐O cells treated with CM from *CYP1B1* silenced adipocytes. Scale bar represents 50 μm. Data are shown as mean ± SD from at least 3 separate experiments. *P*‐values were determined by one‐way ANOVA. *P < 0.05, **P < 0.01, ***P < 0.001 and ****P < 0.0001.

To get better insight into the role of *ADAM12* and *CYP1B1* in the tumor–stroma crosstalk, first, we analyzed the influence of their silencing on RCC cell proliferation. Since A498 cells represent a model of high invasiveness and advanced graded RCC tumors, we performed the functional studies in the less aggressive 786‐O cells [38]. Spheroid formation assay was performed in 786‐O cells following treatment with CMs of *ADAM12*/*CYP1B1* expressing (control) or nonexpressing (silenced) adipocytes from lean, overweight, and obese RCC patients. Spheroids were cultured for different time points following the CM treatment and spheroid diameters were measured. Interestingly, when silencing was performed in lean patients‐derived adipocytes, almost no difference in RCC spheroid size was observed (Fig. 5A). Conversely, *ADAM12* and *CYP1B1* suppression induced a reduction in RCC spheroid size when cultured with CM from overweight patient‐derived adipocytes (Fig. 5B) and even a stronger reduction in RCC spheroid size was generated by CM from obese patients‐derived adipocytes silenced for *ADAM12* and *CYP1B1* (Fig. 5C).

Regarding the comparison between the formed spheroid sizes of the treated 786‐O cells with CMs of lean, overweight, and obese controls, the data show significant differences between obese and lean cases on 2, 7, 9, and 11 days after the CM treatment. On day 11, a statistically significant difference can also be observed between the lean and overweight groups. However, there was no difference between overweight and obese groups in any of the time points (Fig. 5D).

### 3.6 *ADAM12* / *CYP1B1* downregulation in adipocytes reduces the migration of RCC cells

**Fig. 6:**
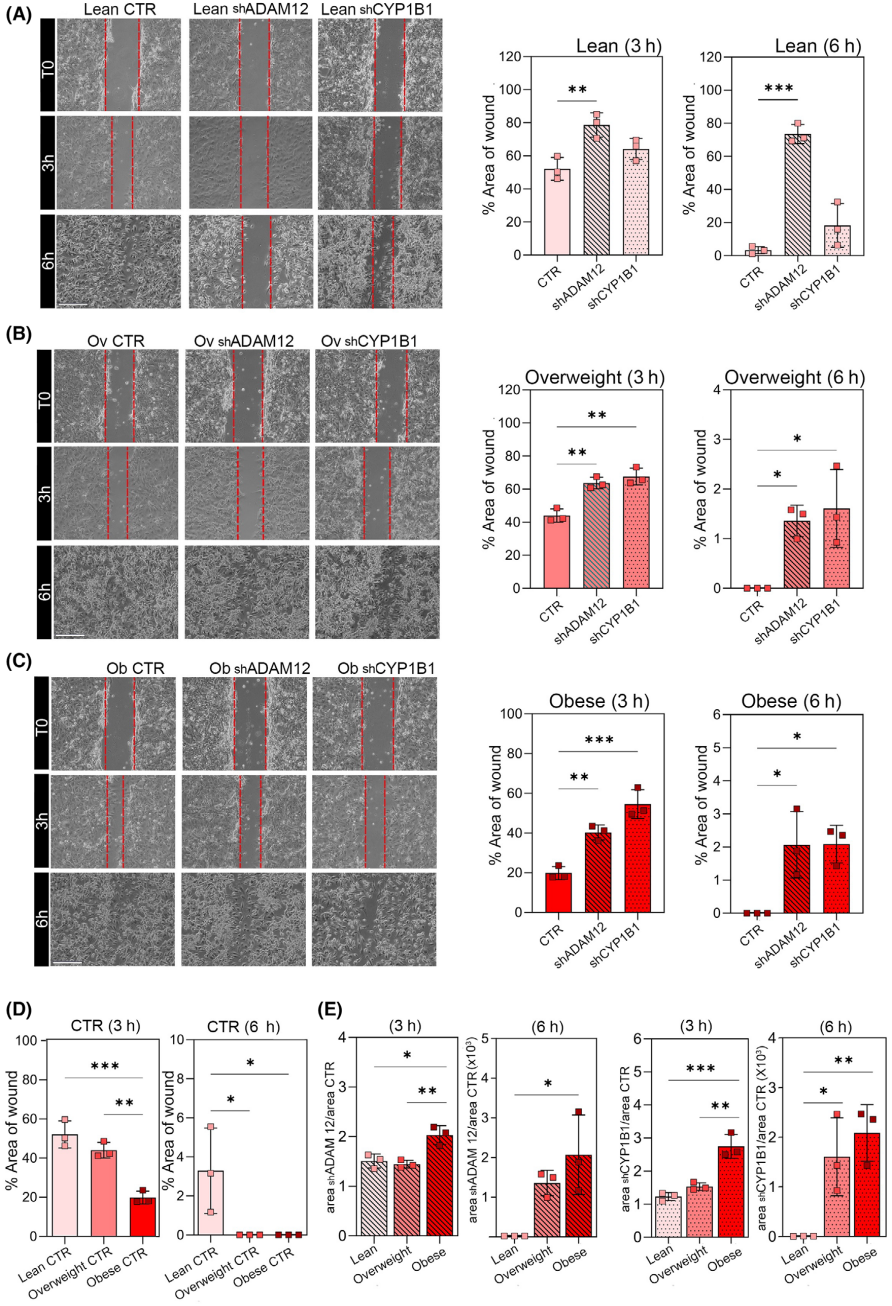
*ADAM12* and *CYP1B1* silencing impacts renal cancer cell (RCC) migration. Wound healing scratch assay for 786‐O cells at time 0 (T0), 3 h, and 6 h after treatment with conditioned media (CMs) of *ADAM12*/*CYP1B1* silenced and non‐silenced adipocytes derived from (A) lean, (B) overweight, and (C) obese RCC patients (*n* = 3). (D) Histograms show the percentage of the areas of wound comparison in RCC cells treated with the CMs collected from adipocytes derived from lean, overweight, and obese patients at the same time points as previously described (*n* = 3). (E) The comparison between the ratio of the shADAM12 or shCYP1B1 areas to control areas of wound in different experimental groups (*n* = 3). Cell migration was measured as described in the Materials and Methods. CTR, 786‐O cells treated with CM from control adipocytes (adipocytes expressing *ADAM12* and *CYP1B1*); Ob, obese; Ov, overweight; shADAM12, 786‐O cells treated with CM from *ADAM12* silenced adipocytes; shCYP1B1, 786‐O cells treated with CM from *CYP1B1* silenced adipocytes. Scale bar represents 200 μm. Data are shown as mean ± SD from at least 3 separate experiments. *P*‐values were determined by one‐way ANOVA. *P < 0.05, **P < 0.01, ***P < 0.001.

We aimed to investigate the effects of CMs, obtained from both *ADAM12*/*CYP1B1* expressing (control), and knocked down (silenced) adipocytes from RCC patients with different stages of BMI on the migration of RCC cells. Thus, we performed the wound healing scratch assay for 786‐O cells treated with CMs from silenced and control differentiated adipocytes of RCC patients categorized as lean, overweight, and obese. The photographs were taken from each case at T0, 3 h, and 6 h after making the scratch, and the free‐cell area was calculated (Fig. 6A–C).

In terms of the comparison between the migration of 786‐O cells treated with CMs of expressing *ADAM12* and *CYP1B1* adipocytes and the ones treated with CM from one of these two genes silenced adipocytes, as can be seen in Fig. 6A–C, knockdown of either of these two genes decreased the ability of the cell migration. However, the difference regarding *CYP1B1* knockdown was not statistically significant in the lean.

Both 3 and 6 h endpoints demonstrated that 786‐O cells treated with the CM of control differentiated adipocytes from RCC obese patients have more ability to migrate than the ones treated with the CM of control differentiated adipocytes from RCC lean patients. At the third hour of the scratch assay, treatment of 786‐O cells with CM of overweight control showed no statistically significant difference with the lean case. While at the sixth hour of the experiment, the scratch related to the control lean condition was still open, both scratches of the RCC cells with control overweight and obese CM treatments were completely closed; therefore, reflecting no difference at the last endpoint between these two conditions (Fig. 6D). As a result, it can be concluded that 786‐O cells treated with CM of the non‐silencing differentiated adipocytes of obese RCC patients showed a higher level of migration than lean RCC patients.

To understand which case of *ADAM12*/*CYP1B1* RNAi (lean, overweight, or obese) has a stronger effect against the migration of 786‐O cells treated with CMs of expressing *ADAM12* and *CYP1B1* adipocytes, the ratios of *ADAM12*/*CYP1B1* knockdown areas to the non‐silencing areas in different experimental groups were calculated. Figure 6E shows the results of the mentioned calculation indicating a stronger effect of CMs of *ADAM12*/*CYP1B1* knockdown in the obese than the lean in the migration ability of 786‐O cells treated with CMs of non‐silencing adipocytes.

### 3.7 Silencing of *ADAM12* and *CYP1B1* potently reduces RCC tumor growth *in vivo*

**Fig. 7:**
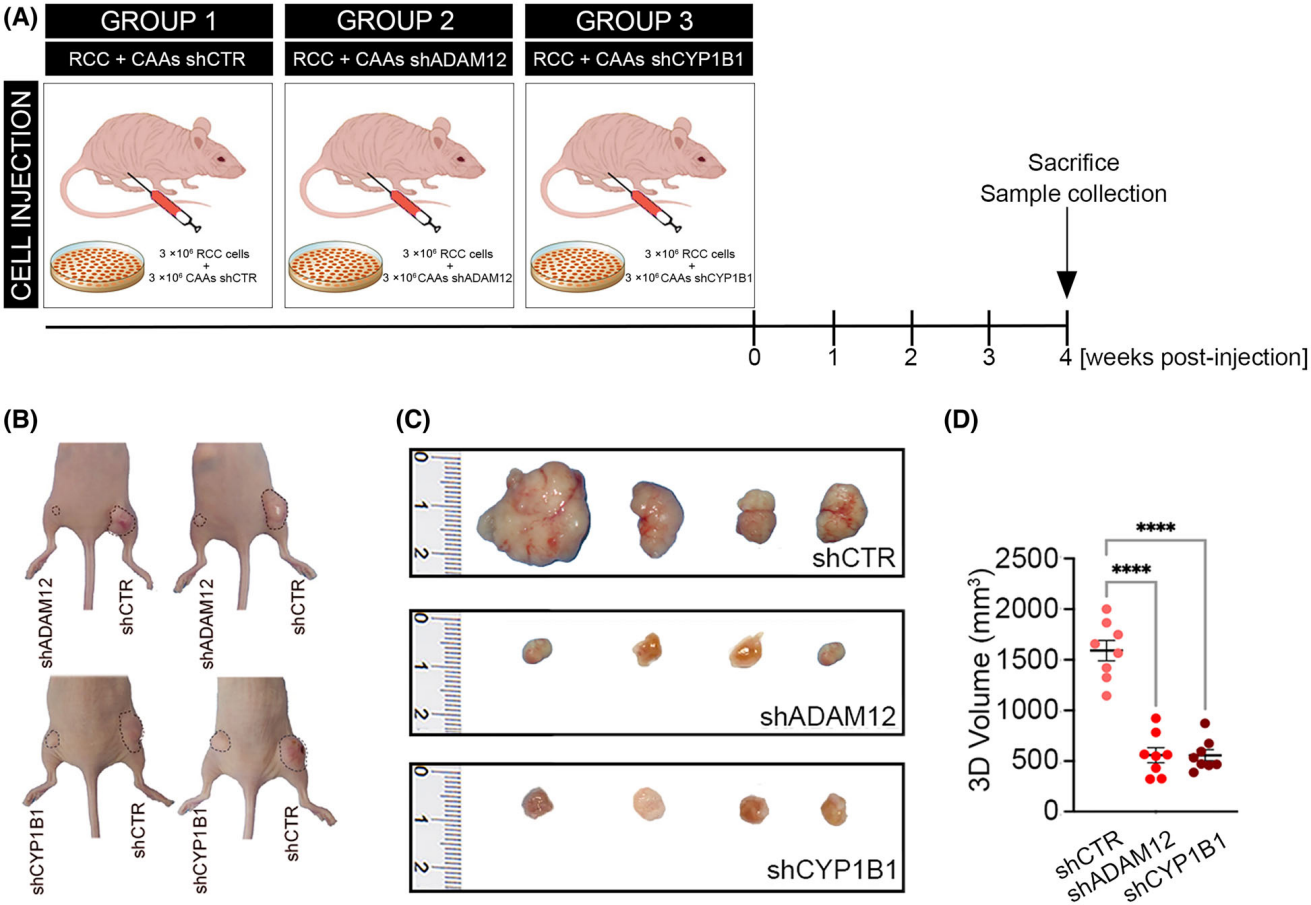
ADAM12 and CYP1B1 silencing reduces the renal cancer cells' (RCC) tumor growth in a xenograft model. (A) Nude mice (*n* = 8) were engrafted subcutaneously with 3 × 10^6^ RCC cancer cells and 3 × 10^6^ cancer‐associated adipocytes (CAAs) transfected with short‐hairpin shCTR, shADAM12, and shCYP1B1. Xenografts and solid tumors were dissected 4 weeks after the implantation and photographed (B, C). (D) 3D volume (mm^3^) of solid tumors derived from nude mice (*n* = 8) engrafted with RCC + CAAs silenced with shADAM12 and shCYP1B1, compared to shCTR. Data are shown as mean ± SD. *P*‐values were determined by one‐way ANOVA. ****P < 0.0001.

Having shown that ADAM12 and CYP1B1 depletion in CAAs drastically attenuates RCC cell growth and migration *in vitro*, we evaluated the role of these two genes in the tumor microenvironment for the oncogenic potential of RCC cells in xenografts, *in vivo*, *n* = 8. To this aim, we subcutaneously co‐injected 3 × 10^6^ RCC cells with 3 × 10^6^ CAAs transfected with shCTR, shADAM12, or shCYP1B1 (Fig. 7A). Interestingly, tumors originating from RCC + shCTR‐CAAs grew rapidly and were palpable 2 weeks after injection. Contrarily, silencing of ADAM12 and CYP1B1 in CAAs generated much smaller RCC tumors, which became palpable 4 weeks after injection (Fig. 7B–D).

These data indicate a functional requirement of ADAM12 and CYP1B1 in peritumoral adipocytes for RCC tumor growth. These data also suggest that effective ADAM12 and CYP1B1 loss leads to a remarkable attenuation of RCC tumorigenic potential *in vivo* and functionally validates our model of a reciprocal regulation of CAAs and RCC in kidney cancer.

## 4 Discussion

Obesity deranges the structure and function of AT such that the gene expression of ATs from obese subjects is different from corresponding nonobese ATs [39]. Obesity supports cancer growth and invasive progression through different mechanisms, among which the promotion of the onset of a chronic inflammatory state and increased cellular oxidative stress are the most relevant. However, the complex relationship between peritumoral AT and cancer cells is yet incompletely understood. Moreover, how differently peritumoral AT from lean versus obese subjects can impact cancer is far to be known. In an effort to understand the molecular differences in peritumoral adipocytes (CAAs) from lean versus obese subjects, we isolated CAAs from RCC patients stratified into three groups according to BMI. By performing an RNA‐seq array on the three groups of CAAs, we found that the most altered genes were involved in the tumor–stroma crosstalk (i.e., genes belonging to the category of extracellular matrix organization and angiogenesis).

### 4.1 The peritumoral adipocytes in the tumor microenvironment

Different stromal cell types can modify the growth and progression of tumors. In recent years, interest has extended to adipocytes and adipose tissue‐derived mesenchymal stem cells. The tight interaction between cancer and adipose cells fosters the reprogramming of adipocytes, converted into CAAs. These in turn facilitate tumor growth and progression by releasing adipokines, growth factors, and metabolites. Understanding the crosstalk between CAAs and tumoral cells, and how obesity exacerbates the potency of CAAs in promoting tumorigenesis is essential in the prevention of tumor progression. Here, we report that the CAAs in renal cancer are potently affected by the body weight of the patients. Notably, CAAs from lean patients have higher expression of the ‘good’ adipokine adiponectin, in line with its anticancer role [40]. Conversely, in obese patients CAAs show a reduction of adiponectin and a drastic increase in leptin expression, confirming its pro‐tumorigenic value [41]. Indeed, in the dynamic crosstalk between AT and tumor cells, the leptin/adiponectin ratio has a key role in determining the risk of cancer formation and progression through low‐grade chronic inflammation pathways. However, still, the molecular modes by which obese‐derived AT influences tumorigenesis are not completely known. Our study confirms that the peritumoral adipocytes from obese subjects are characterized by an altered leptin/adiponectin ratio. Moreover, the expression of pro‐inflammatory cytokines increases linearly with the subjects BMI. Furthermore, by performing experiments of conditioned media (CMs) among renal cancer cells and CAAs along with *in vivo* xenograft experiments, we found that obese‐derived adipocytes stimulate RCC growth and migration when compared to lean‐derived CAAs. These observations add new solid insights into the role of obesity in cancer, pointing to a crucial role in the TME and its paracrine role in cancer promotion.

### 4.2 The expression of *ADAM12* and *CYP1B1* in the TME affects RCC cancer

The novel aspect of our work is that we not only found two genes (*ADAM12* and *CYP1B1*) upregulated in CAAs of renal cancer but also, we observed that their expression correlates with BMI and is tightly linked with the overweight‐to‐obese degree of patients. Indeed, we sought to investigate the roles of *ADAM12* and *CYP1B1* in our work since they: (a) are among the most significantly upregulated genes in CAAs from overweight and obese compared to the lean subjects; (b) their expression has been proved to foster tumorigenesis and have a crucial role in tumor microenvironment; (c) *ADAM12* and *CYP1B1* play crucial roles in both, adipogenesis [42, 43] and tumorigenesis [35, 44]. Thereby, our findings add further relevance for these two genes in fat‐driven cancerogenesis.

ADAM12 is part of the matrix metalloproteinase‐related protein family and participates in the proteolytic processing of other transmembrane proteins, regulating transcription, cell‐signaling pathways, apoptosis, cell‐cycle progression, and cell adhesion [24, 45, 46, 47]. It has been also found upregulated in different types of tumors [48, 49, 50]. Furthermore, *ADAM12* hypomethylation was associated with a worse outcome in triple‐negative breast cancer (TNBC), in peritumoral regions and circulating tumoral cells [51]. Moreover, it was observed that *ADAM12* silencing reduced TNBC cell proliferation and migration and improved doxorubicin sensitivity in TNBC cells, indicating that *ADAM12* is a potential therapeutic target and its hypomethylation could be a poor outcome biomarker in TNBC [51]. Due to its role as disintegrin and metalloproteinase, its relevance in the TME and mediating the crosstalk between cancer cells and the surrounding milieu can be critical in supporting tumor invasiveness and progression. Our work shed new insights into the role of *ADAM12* in CAAs demonstrating its overexpression in the peritumoral AT and *CAAs. Notably*, we confirmed that *ADAM12* silencing reduces the proliferation and migration of RCC, proving its role in tumor microenvironment. These observations suggest that ADAM12 can mechanistically be involved in TME and that, its inhibition can be exploited to attenuate the aggressiveness in kidney cancer patients.

CYP1B1 belongs to the cytochrome P450 (CYPs) superfamily of enzymes and is a key player in the oxidative metabolism of exogenous and endogenous compounds, as well as in the detoxification of pre‐carcinogens, such as polycyclic aromatic hydrocarbons and estrogens [52]. *CYP1B1* is overexpressed in cancer [53, 54]. The ability of CYP1B1 in promoting the activation of potential carcinogens increases its relevance in DNA mutagenesis. For instance, it catalyzes the conversion of estrogens to quinone forms, which in turn bind the DNA and enhance the cancer risk in several organs, such as the brain, breast, and ovary [55, 56]. Our data indicate that *CYP1B1* is also expressed in the CAAs and that it fosters AT‐dependent cancer promotion. The role of CYP1B1 in adipose tissue is not new, since it has been reported in mesenchymal stromal cells during adipogenesis in parallel with PPARγ, a critical transcriptional factor in adipogenic process [42]. Importantly, previous works have demonstrated that CYP1B1 is overexpressed in breast cancer cells [57] and that leptin upregulates CYP1B1 mRNA and protein expression in breast cancer [58], thus suggesting that increase in CYP1B1 expression may be one of the mechanisms for the pro‐tumoral activity of leptin. Moreover, *CYP1B1* deficiency attenuated HFD‐induced obesity and improved glucose tolerance due to the reduced expression of different adipogenic genes such as *PPARγ*, *CD36*, *FAS*, and *SCD*‐1 and increased expression of genes involved in lipolysis [59]. Indeed, *CYP1B1* is upregulated during the adipocyte differentiation, when *CYP1B1* increases *PPARγ* expression [42]. Thus, considering that *PPARγ* activation induces the expression of leptin [60, 61], in turn, a reciprocal crosstalk between leptin and CYP1B1 may contribute to the effects of both leptin and CYP1B1 in tumorigenesis, fat metabolism, and energy homeostasis.

Finally, since we have shown that RCC tumors with high *ADAM12* and *CYP1B1* expression are characterized by enhanced proliferation and migration of cancer cells, it is reasonable to speculate that monitoring the invasiveness and the potential metastatic evolution of patients with the highest expression of these two genes will be an unmet goal of the follow‐up in the future.

## 5 Conclusion

In conclusion, our results demonstrate that *ADAM12* and *CYP1B1* are highly expressed in CAAs of kidney cancer and that their expression linearly follows the body weight. Enhanced *ADAM12* and *CYP1B1* expression promotes tumorigenesis of renal cancer since their silencing reduces the tumor growth and cancer cell proliferation and migration. Taken together, our results confirm the key role of peritumoral adipose tissue in exacerbating the aggressiveness of kidney cancer. Thus, not only ADAM12 and CYP1B1 can be seen as prognostic markers in RCC, but we also propose that interfering with the identified genes as well as hampering adiposity is an unmet adjuvant strategy in cancer therapy. Our work points to a critical role for these two proteins in the tumor–stroma crosstalk, especially considering the worldwide pandemic of obesity, and its impact on cancer.

## Conflict of interest

The authors declare no conflict of interest.

## Author contributions

ST, CM, AN, AGC, SS, LA, FR, MM, and EDC performed *in vitro* and *in vivo* experiments. CM and AN performed the immunofluorescence analysis; ST, CM, and AN performed bioinformatic analysis, analyzed the results, and provided scientific interpretations; FDG and FCapone provided human samples; CI, MD, and FCrocetto supervised the experiments, analyzed the results, and provided scientific interpretations; and MD designed the overall study, supervised the experiments, and analyzed the results. ST and MD wrote the paper. All authors discussed the results and provided input on the manuscript.

## Supporting information


**Fig. S1.** Strategy for the silencing of ADAM12 expression. (A) Cloning strategies for the generation of ADAM12 shRNA expression vectors. (B‐D) Electropherograms of three different ADAM12 shRNA sequences cloned into pcRNAi vector.
**Fig. S2.** Strategy for the silencing of CYP1B1 expression. (A‐C) Electropherograms of three different CYP1B1 shRNA sequences cloned into pcRNAi vector.
**Fig. S3.** mRNA expression ratio of tumor suppressor genes in tumor to healthy kidney tissues among lean, overweight, and obese RCC patients.
**Fig. S4.** Mature adipocytes obtained from ASCs by a 15‐day adipogenesis protocol. lean, overweight, and obese differentiated adipocytes stained with Oil Red O. Each red spot represents an oil droplet. Scale bar represents 50 μm.
**Fig. S5.** Leptin/adiponectin mRNA ratio in the differentiated adipocytes derived from lean, overweight, and obese RCC patients.
**Fig. S6.** Soluble molecules concentration in the Conditioned Media of ADAM12/CYP1B1 expressing and silenced differentiated adipocytes. A) IL‐10, B) IL‐13, C) IL‐15, D) IL‐1β, and E) IL‐4 concentration (pg/mL) in CMs of ADAM12 and CYP1B1 expressing and knocked down adipocytes derived from lean, overweight, and obese RCC patients. Abbreviations: CTR, CM from control adipocytes (adipocytes expressing ADAM12 and CYP1B1); shADAM12, CM from ADAM12 silenced adipocytes; shCYP1B1, CM from CYP1B1 silenced adipocytes; Ov, overweight; Ob, obese. *P < 0.05, **P < 0.01.

## Data Availability

The clinical data from patients are provided in Table 1. Primer list is provided in Table 2. All data that support the findings of this study are available in the article, Supporting Information, or from the corresponding author upon reasonable request.
