# Supplementary material for: Obesity alters the fitness of peritumoral adipose tissue, exacerbating tumor invasiveness in renal cancer through the induction of ADAM12 and CYP1B1
Source: Mol Oncol. 2025 Jan 13;19(6):1612–32. doi: 10.1002/1878-0261.13782 (PMC12161473; doi:10.1002/1878-0261.13782)
Supplement: Supplementary file 1 — Fig. S1. Strategy for the silencing of ADAM12 expression. (A) Cloning strategies for the generation of ADAM12 shRNA expression vectors. (B‐D) Electropherograms of three different ADAM12 shRNA sequences cloned into pcRNAi vector. Fig. S2. Strategy for the silencing of CYP1B1 expression. (A‐C) Electropherograms of three different CYP1B1 shRNA sequences cloned into pcRNAi vector. Fig. S3. mRNA expression ratio of tumor suppressor genes in tumor to healthy kidney tissues among lean, overweight, and obese RCC patients. Fig. S4. Mature adipocytes obtained from ASCs by a 15‐day adipogenesis protocol. lean, overweight, and obese differentiated adipocytes stained with Oil Red O. Each red spot represents an oil droplet. Scale bar represents 50 μm. Fig. S5. Leptin/adiponectin mRNA ratio in the differentiated adipocytes derived from lean, overweight, and obese RCC patients. Fig. S6. Soluble molecules concentration in the Conditioned Media of ADAM12/CYP1B1 expressing and silenced differentiated adipocytes. A) IL‐10, B) IL‐13, C) IL‐15, D) IL‐1β, and E) IL‐4 concentration (pg/mL) in CMs of ADAM12 and CYP1B1 expressing and knocked down adipocytes derived from lean, overweight, and obese RCC patients. Abbreviations: CTR, CM from control adipocytes (adipocytes expressing ADAM12 and CYP1B1); shADAM12, CM from ADAM12 silenced adipocytes; shCYP1B1, CM from CYP1B1 silenced adipocytes; Ov, overweight; Ob, obese. *P < 0.05, **P < 0.01. [file MOL2-19-1612-s001.pdf]

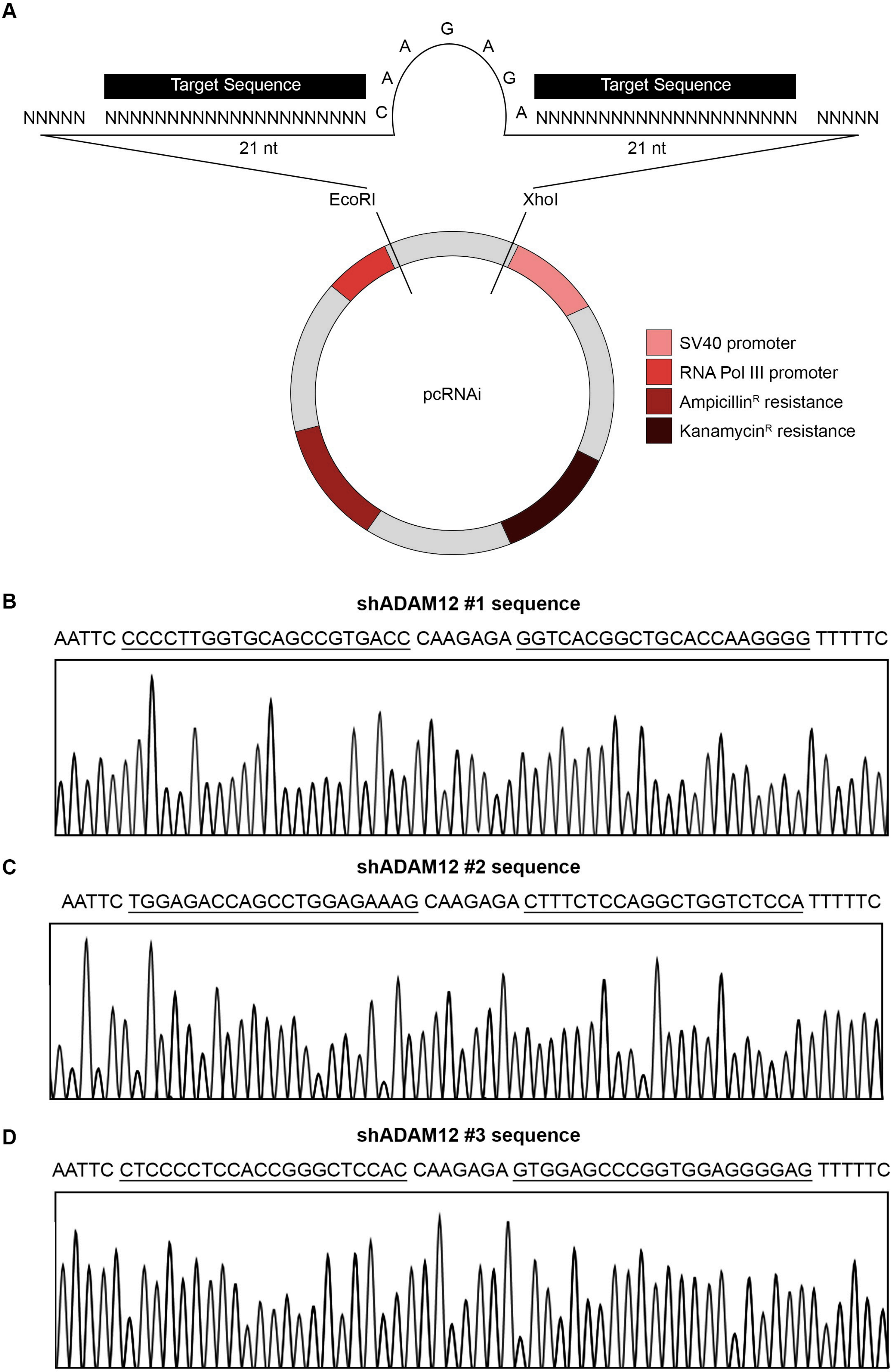

Supplemental Figure 1

A

shCYP1B1 #1 sequence

AATTC <u>CGGCGGCCGCAGCATGGCTTT</u> CAAGAGA <u>AAAGCCATGCTGCGGCCGCCG</u> TTTTTC

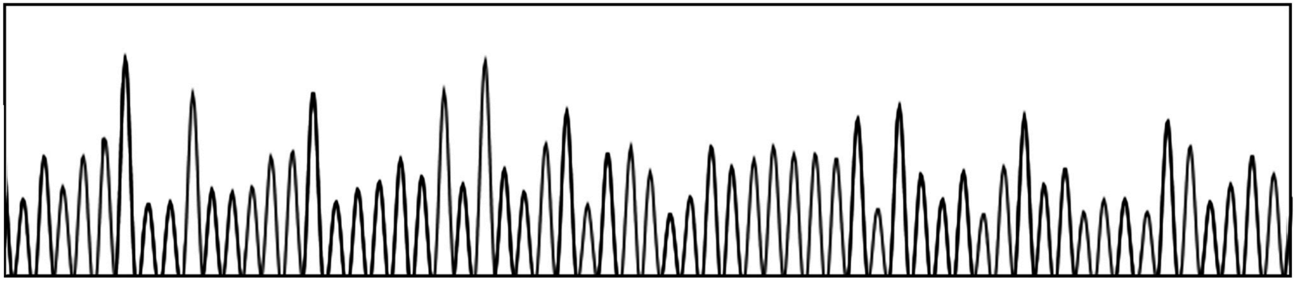

B

shCYP1B1 #2 sequence

AATTC <u>GGGCCGCCCCCGCGACATGA</u> CAAGAGA <u>TCATGTCGCGGGGGCGGCCC</u> TTTTTC

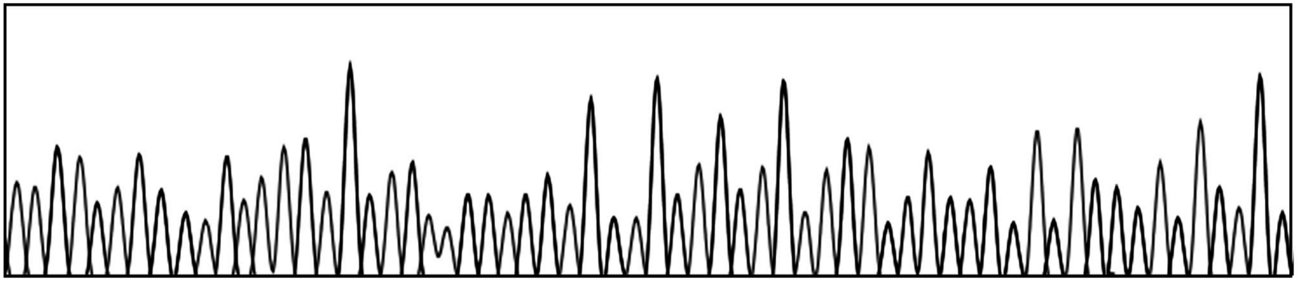

C

shCYP1B1 #3 sequence

AATTC <u>TGTCCTGGCCTTCCTTTATGA</u> CAAGAGA <u>TCATAAAGGAAGGCCAGGACA</u> TTTTTC

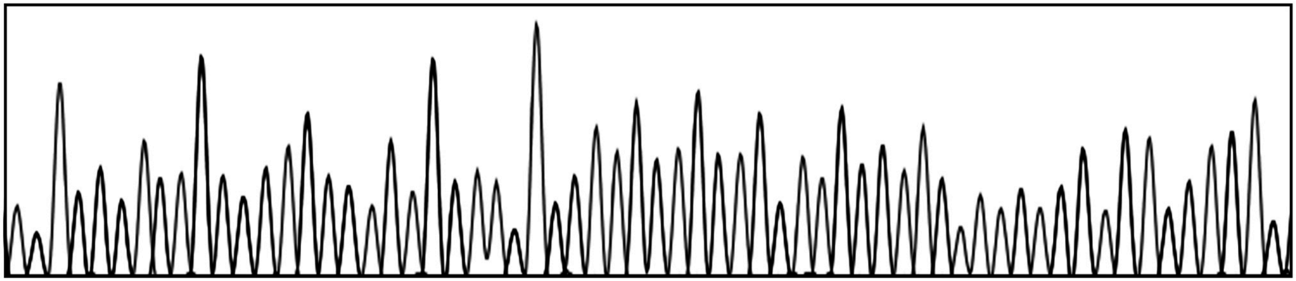

Supplemental Figure 2

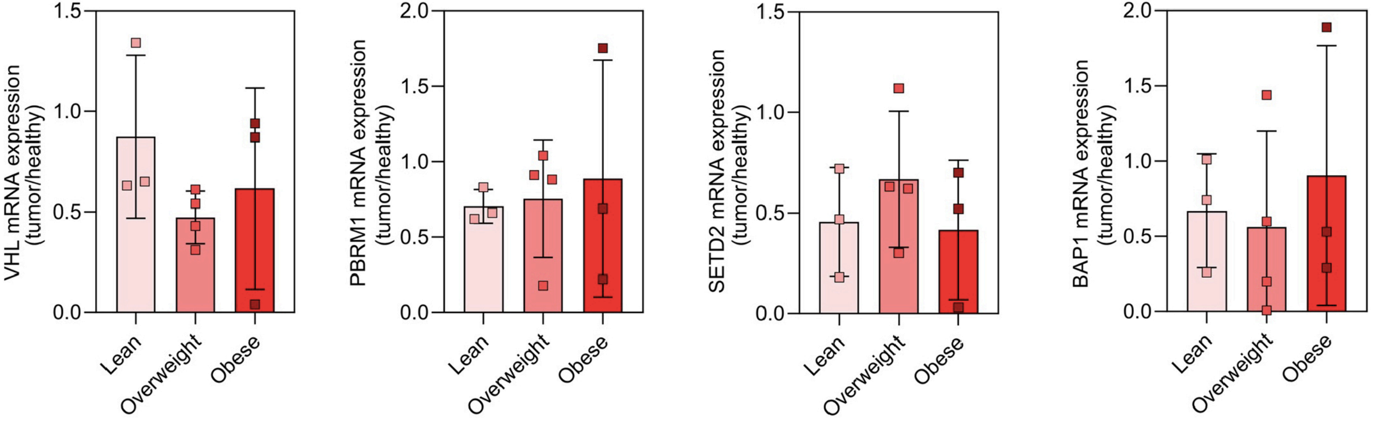

Supplemental Figure 3

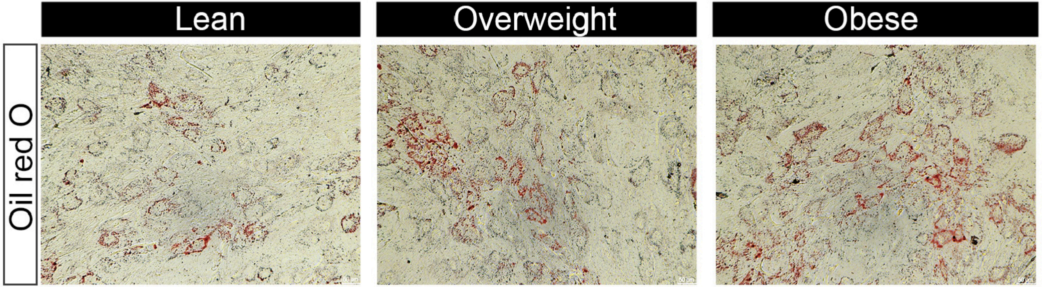

Supplemental Figure 4

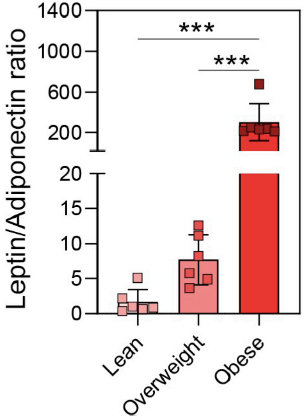

Supplemental Figure 5

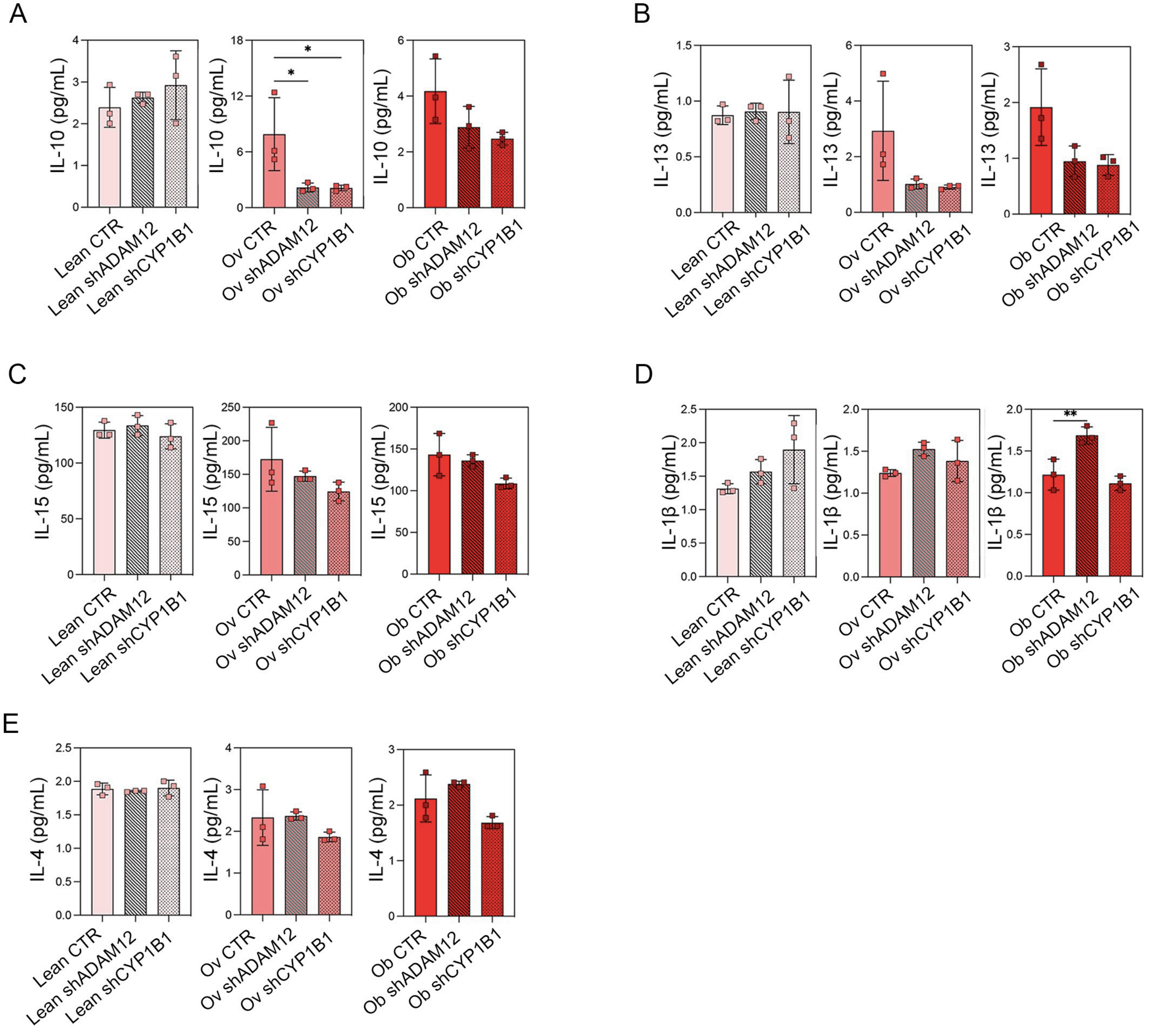

Supplemental Figure 6
